# The genome sequence of Cory’s shearwater, *Calonectris borealis *(Cory, 1881)

**DOI:** 10.12688/wellcomeopenres.23354.1

**Published:** 2024-11-12

**Authors:** Guillem Izquierdo Arànega, Joan Ferrer Obiol, Raül Ramos Garcia, Marta Riutort León, Julio Rozas Liras, Jacob González-Solís Bou, Wellcome Sanger Institute Tree of Life Management, Samples and Laboratory team, Wellcome Sanger Institute Scientific Operations: Sequencing Operations, Wellcome Sanger Institute Tree of Life Core Informatics team, Tree of Life Core Informatics collective, Darwin Tree of Life Consortium

**Keywords:** Calonectris borealis, Cory’s shearwater, genome sequence, chromosomal, Procellariiformes

## Abstract

We present a genome assembly from an individual female *Calonectris borealis* (Cory’s shearwater; Chordata; Aves; Procellariiformes; Procellariidae). The haplotype-resolved assembly contains two haplotypes with total lengths of 1,366.19 megabases and 1,211.47 megabases, respectively. Most of the assembly for haplotype 1 is scaffolded into 41 chromosomal pseudomolecules, including the Z and W sex chromosomes. Haplotype 2 has 39 autosomes. The mitochondrial genome has also been assembled and is 19.95 kilobases in length.

## Species taxonomy

Eukaryota; Opisthokonta; Metazoa; Eumetazoa; Bilateria; Deuterostomia; Chordata; Craniata; Vertebrata; Gnathostomata; Teleostomi; Euteleostomi; Sarcopterygii; Dipnotetrapodomorpha; Tetrapoda; Amniota; Sauropsida; Sauria; Archelosauria; Archosauria; Dinosauria; Saurischia; Theropoda; Coelurosauria; Aves; Neognathae; Neoaves; Aequornithes; Procellariiformes; Procellariidae; *Calonectris*; *Calonectris borealis* (Cory, 1881) (NCBI:txid1323832).

## Background

**Figure 1.**
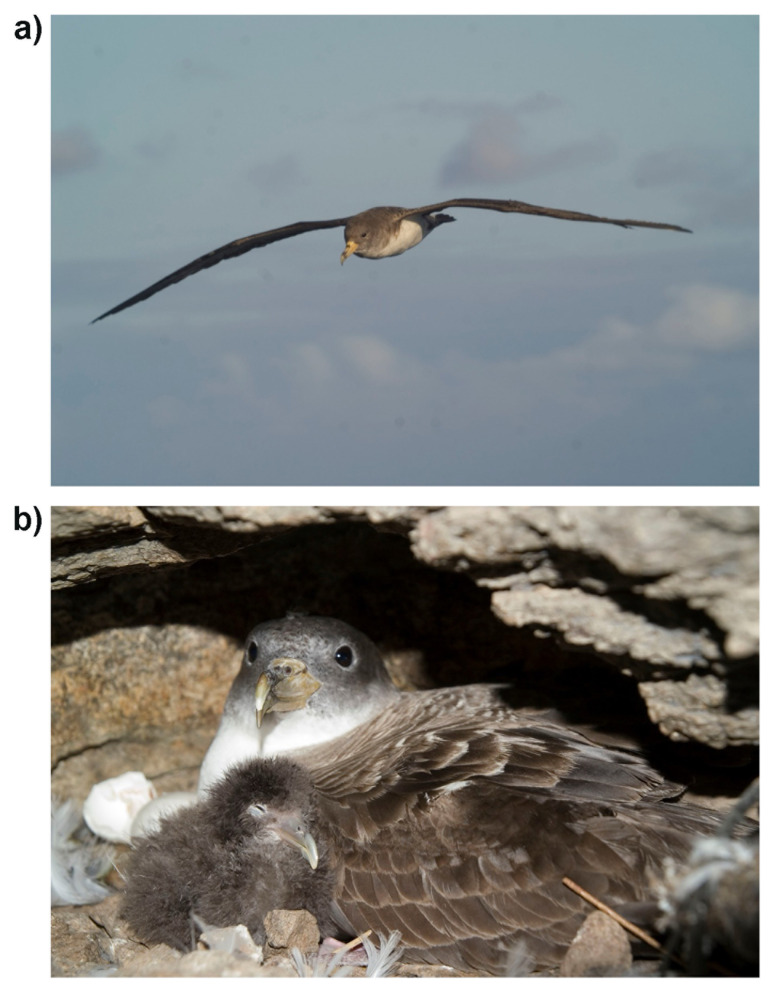
( **a**) Adult Cory’s shearwater approaching the breeding colony of Selvagens Islands (Portugal) at sunset. Credit: Raül Ramos. ( **b**) A recently hatched chick and one of its parents in a burrow on the colony of Veneguera, Gran Canaria (Spain). The specimen used for the genome assembly was sampled in the same colony. Credit: Raül Ramos.

The Cory’s shearwater ( *Calonectris borealis*) ( Figure 1a) is a medium-sized pelagic seabird that acts as an apex predator across the temperate Atlantic Ocean. The species breeds colonially on sea cliffs of islands and islets in the Macaronesian archipelagos: the Canary Islands, Madeira, and the Azores. There are also smaller colonies on islets off the coast of the Iberian Peninsula ( del Hoyo *et al.*, 2020). After breeding, most populations embark on a trans-equatorial migration to the Southern Hemisphere, where they winter off the coast of Africa and South America, primarily on the Benguela, Agulhas and Brazil currents ( De Felipe *et al.*, 2019). In the UK, the species is a regular migrant during late summer (July to October), mostly off headlands in south-western England.

During the breeding period, from March to October, Cory’s shearwaters visit their colonies at night. These monogamous birds raise a single chick in underground burrows and caves ( Figure 1b). When at sea, the species wanders over oceanic waters near the continental shelf, where it feeds on pelagic fish and cephalopods ( Brooke, 2004).

During the last decades, the species has suffered population declines in several colonies linked to anthropogenic activities ( Fontaine *et al.*, 2011). In their breeding colonies, shearwaters are particularly vulnerable to predation by introduced rats and cats, and at sea, they are impacted by fisheries bycatch ( Soriano-Redondo *et al.*, 2016), which kills hundreds of thousands of seabirds globally each year through incidental capture ( Dias *et al.*, 2019). In addition, during the 20th century, the species faced a serious threat from poaching for its meat and down, with some colonies recording the harvesting of up to 12,000 birds per year ( Lopez-Darias *et al.*, 2011). More recently, collisions caused by light pollution in coastal areas have become an important concern ( Rodríguez *et al.*, 2022). Despite its vulnerability to these threats and its unknown demographic trends, the species is listed as Least Concern by the IUCN Red List.

The Cory’s shearwater is a model species for studies on movement ecology and seabird migration ( González-Solís *et al.*, 2007; Ramos & González-Solís, 2012). Specifically, these studies have elucidated the differences in migratory behaviour between the Cory's shearwater and its sister taxon, the Scopoli’s shearwater ( *C. diomedea*). These two species occasionally hybridise in the Western Mediterranean ( De Felipe *et al.*, 2019; Gómez-Díaz *et al.*, 2009). The generation of a chromosome-level reference genome for the species is a key step for combining genomic data with large tracking databases to investigate the genetic basis for the differences in migratory behaviour among Cory’s shearwater populations and between closely related species. It may also enable further whole-genome resequencing studies to characterise hybridisation patterns across the contact zone between Cory’s and Scopoli’s shearwaters.

## Genome sequence report

The genome of a female *Calonectris borealis* was sequenced using Pacific Biosciences single-molecule HiFi long reads, generating a total of 83.84 Gb (gigabases) from 6.46 million reads, providing approximately 66-fold coverage. Primary assembly contigs were scaffolded with chromosome conformation Hi-C data, which produced 68.13 Gb from 451.21 million reads. Specimen and sequencing details are provided in Table 1.

**Table 1.** Specimen and sequencing data for *Calonectris borealis*.

Project information			
**Study title**	*Calonectris borealis* (Cory's shearwater)		
**Umbrella BioProject**	PRJEB75561		
**Species**	*Calonectris borealis*		
**BioSample**	SAMEA114294356		
**NCBI taxonomy ID**	1323832		
Specimen information			
**Technology**	**ToLID**	**BioSample** **accession**	**Organism part**
**PacBio long read sequencing**	bCalBor7	SAMEA114294382	Blood
**Hi-C sequencing**	bCalBor7	SAMEA114294382	Blood
Sequencing information			
**Platform**	**Run** **accession**	**Read count**	**Base count (Gb)**
**Hi-C Illumina NovaSeq 6000**	ERR13093660	8.18e+08	123.5
**Hi-C Illumina NovaSeq X**	ERR13093661	4.51e+08	68.13
**PacBio Sequel IIe**	ERR13071485	2.93e+05	2.55
**PacBio Revio**	ERR13071484	6.46e+06	83.84

Both haplotypes were combined for assembly. Manual assembly curation corrected 115 missing joins or mis-joins, increasing the assembly length by 4.2% and reducing the scaffold number by 6.08%.

**Figure 2.**
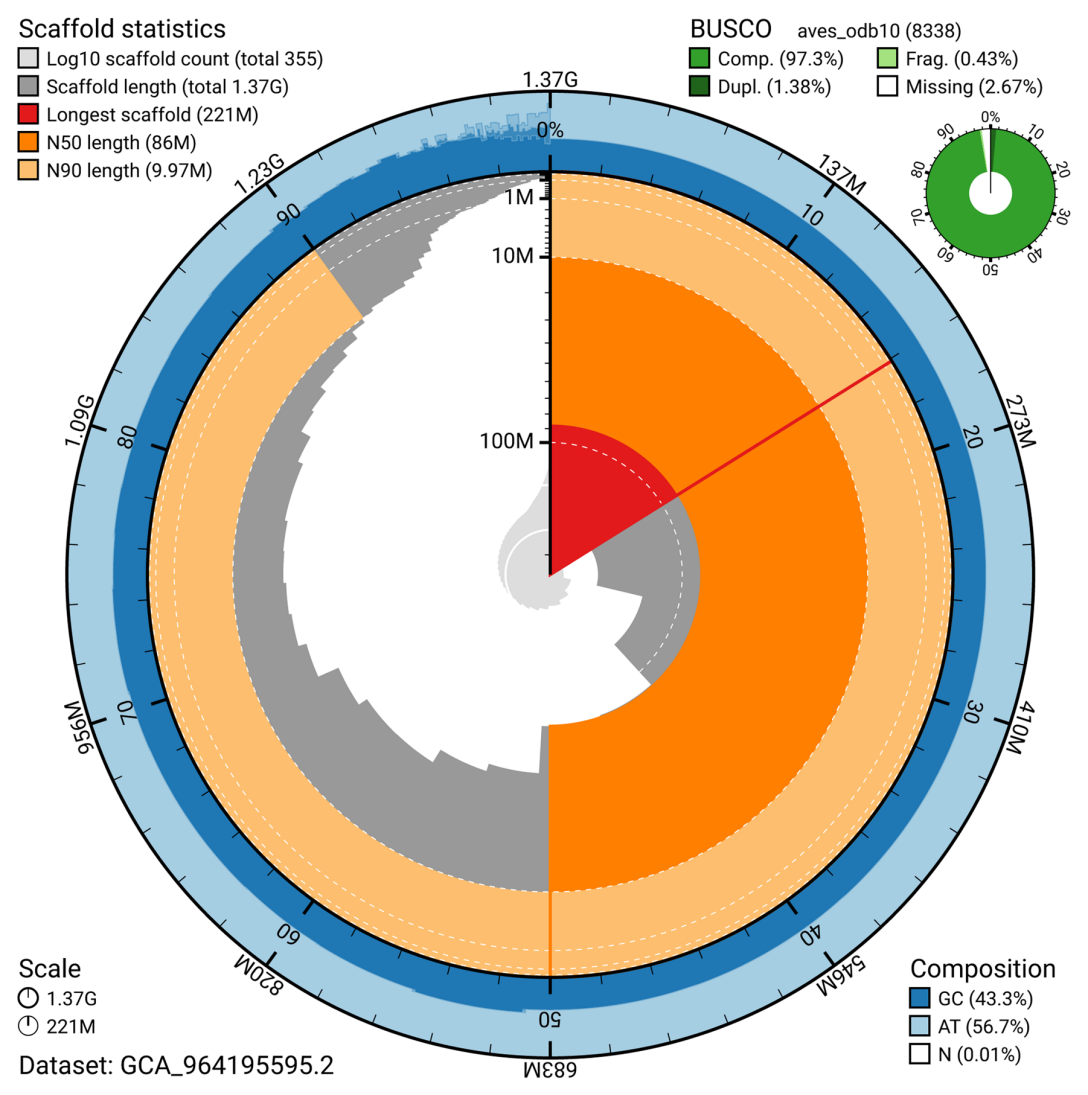
Genome assembly of *Calonectris borealis*, bCalBor7.hap1.2: metrics The BlobToolKit snail plot shows N50 metrics and BUSCO gene completeness. The main plot is divided into 1,000 bins around the circumference with each bin representing 0.1% of the 1,366,205,003 bp assembly. The distribution of sequence lengths is shown in dark grey with the plot radius scaled to the longest sequence present in the assembly (221,108,703 bp, shown in red). Orange and pale-orange arcs show the N50 and N90 sequence lengths (86,023,716 and 9,973,482 bp), respectively. The pale grey spiral shows the cumulative sequence count on a log scale with white scale lines showing successive orders of magnitude. The blue and pale-blue area around the outside of the plot shows the distribution of GC, AT and N percentages in the same bins as the inner plot. A summary of complete, fragmented, duplicated and missing BUSCO genes in the aves_odb10 set is shown in the top right. An interactive version of this figure is available at https://blobtoolkit.genomehubs.org/view/GCA_964195595.2/dataset/GCA_964195595.2/snail.

**Figure 3.**
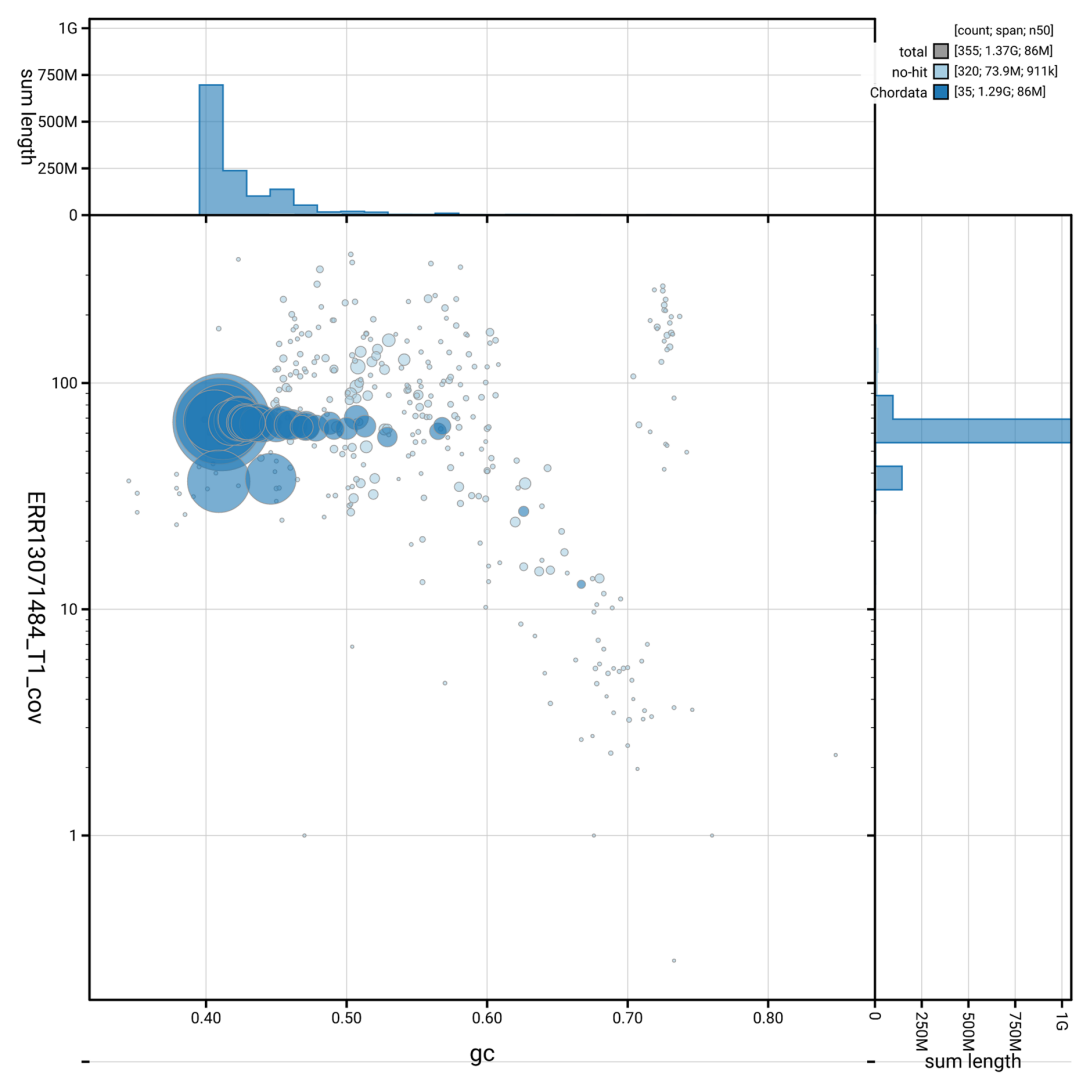
Genome assembly of *Calonectris borealis*: Blot plot of base coverage in the raw data against GC proportion for sequences in bCalBor7.hap1.2. Sequences are coloured by phylum. Circles are sized in proportion to sequence length. Histograms show the distribution of sequence length sum along each axis. An interactive version of this figure is available at https://blobtoolkit.genomehubs.org/view/GCA_964195595.2/dataset/GCA_964195595.2/blob.

**Figure 4.**
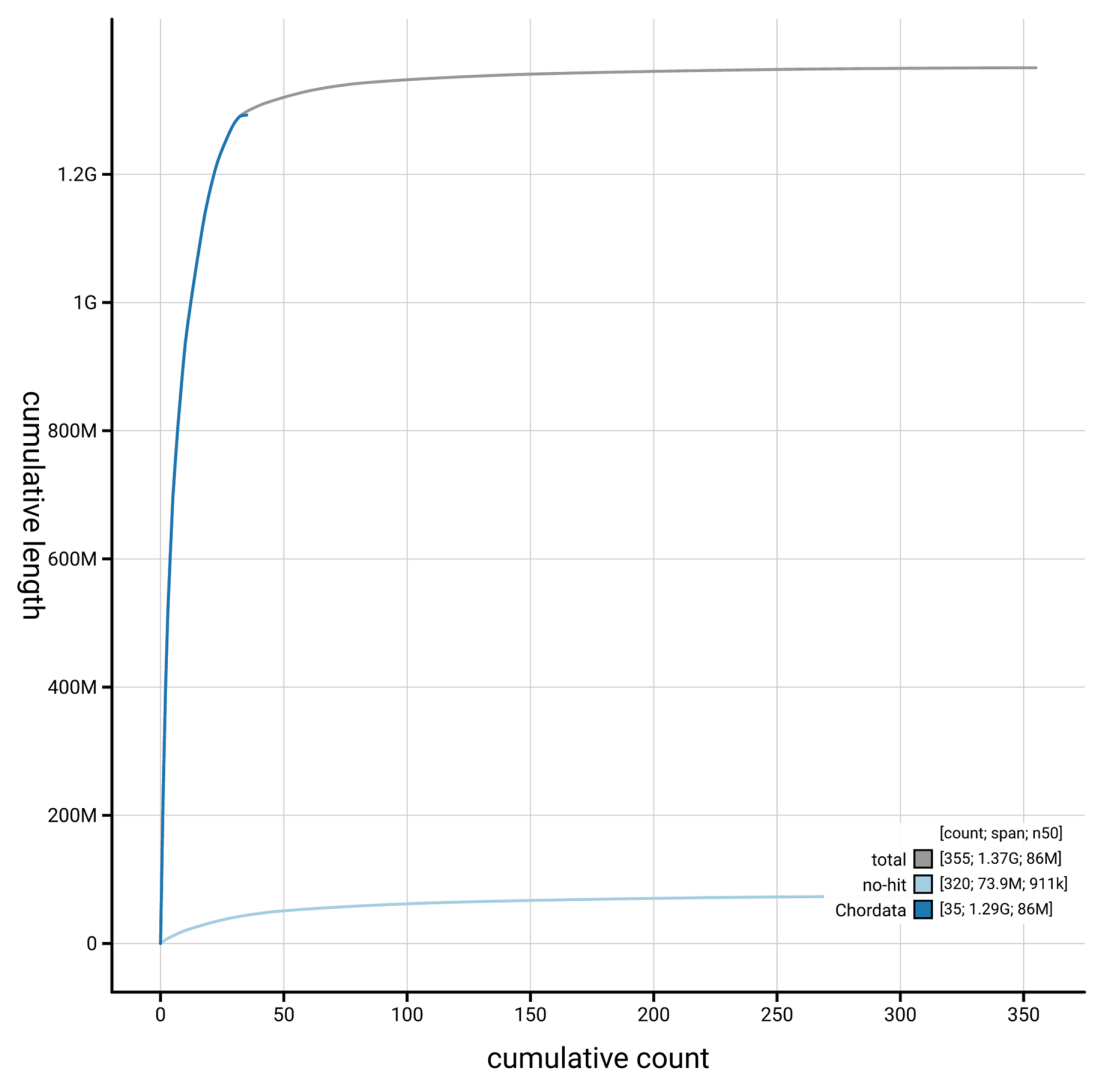
Genome assembly of *Calonectris borealis* bCalBor7.hap1.2: BlobToolKit cumulative sequence plot. The grey line shows cumulative length for all scaffolds. Coloured lines show cumulative lengths of scaffolds assigned to each phylum using the buscogenes taxrule. An interactive version of this figure is available at https://blobtoolkit.genomehubs.org/view/GCA_964195595.2/dataset/GCA_964195595.2/cumulative.

**Figure 5.**
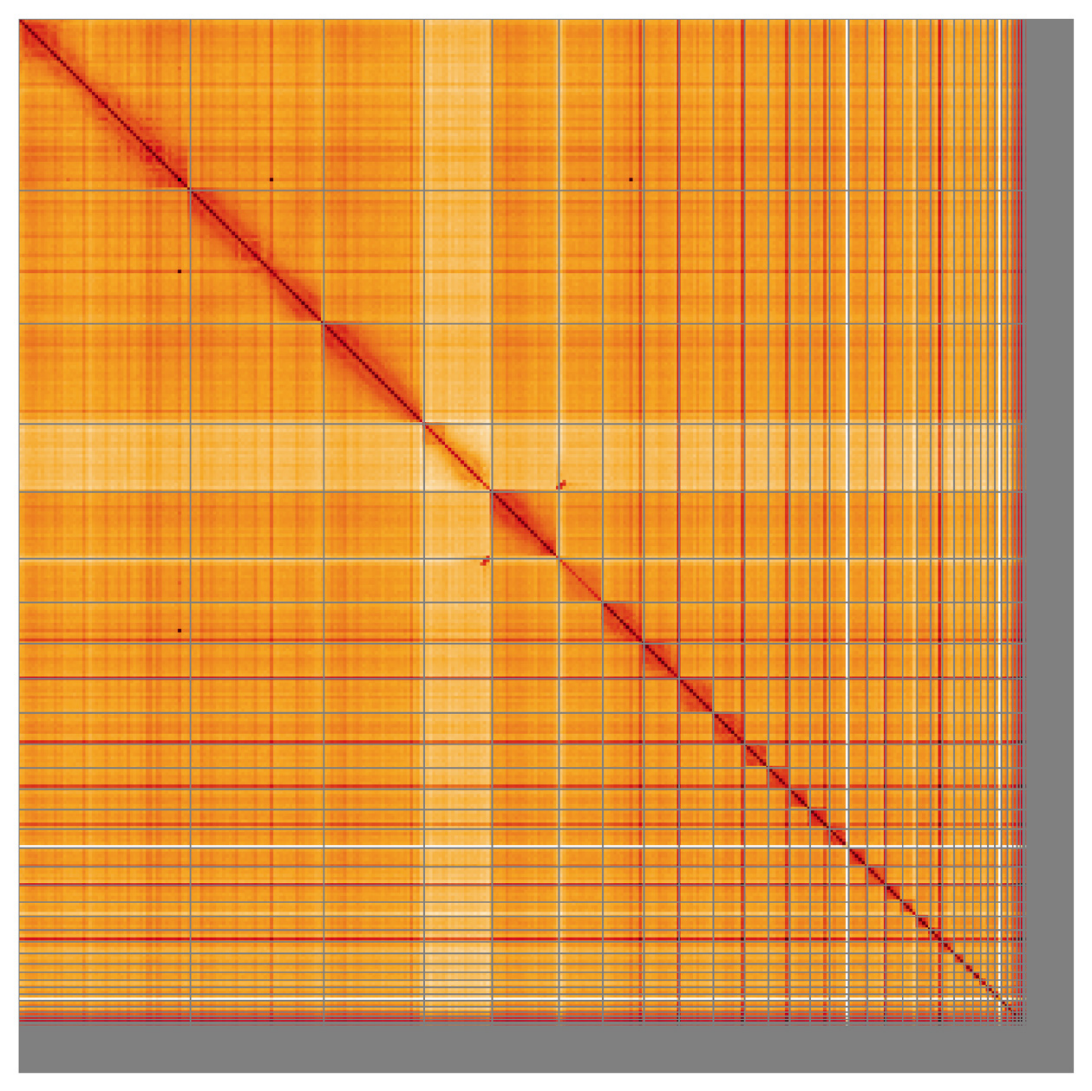
Genome assembly of *Calonectris borealis* bCalBor7.hap1.2: Hi-C contact map of the bCalBor7.hap1.2 assembly, visualised using HiGlass. Chromosomes are shown in order of size from left to right and top to bottom. An interactive version of this figure may be viewed at https://genome-note-higlass.tol.sanger.ac.uk/l/?d=AejNTOCAQHGrm7r429cL-A.

The final assembly for haplotype 1 has a total length of 1,366.20 Mb in 354 sequence scaffolds, with 525 gaps, and a scaffold N50 of 86.0 Mb ( Table 2). The snail plot in Figure 2 provides a summary of the assembly statistics, while the distribution of assembly scaffolds on GC proportion and coverage is shown in Figure 3. The cumulative assembly plot in Figure 4 shows curves for subsets of scaffolds assigned to different phyla. Most (95.07%) of the assembly sequence was assigned to 41 chromosomal-level scaffolds, representing 39 autosomes and the Z and W sex chromosomes. Chromosome-scale scaffolds confirmed by the Hi-C data are named in order of size ( Figure 5; Table 3). The mitochondrial genome was also assembled and can be found as a contig within the multifasta file of the genome submission.

**Table 2.** Genome assembly data for *Calonectris borealis*, haplotype 1 and haplotype 2.

Genome assembly	Haplotype 1	Haplotype 2
Assembly name	bCalBor7.hap1.2	bCalBor7.hap2.2
Assembly accession	GCA_964195595.2	GCA_964196065.2
Assembly level	chromosome	chromosome
Span (Mb)	1,366.19	1,211.47
Number of contigs	879	759
Number of scaffolds	354	281
Longest scaffold (Mb)	221.11	220.95
Assembly metrics (Benchmark) *	Haplotype 1	Haplotype 2
Contig N50 length (≥ 1 Mb)	4.1 Mb	4.1 Mb
Scaffold N50 length (= chromosome N50)	86.0 Mb	86.7 Mb
Consensus quality (QV) (≥ 40)	66.4	66.6
*k-*mer completeness (≥ 95%)	100.0%	100.0%
BUSCO ** (S > 90%; D < 5%)	C:97.3%[S:95.9%,D:1.4%], F:0.5%,M:2.2%,n:8,338	C:93.0%[S:92.7%,D:0.3%], F:0.5%,M:6.5%,n:8,338
Percentage of assembly mapped to chromosomes (≥ 90%)	95.07%	94.91%
Sex chromosomes (localised homologous pairs)	W and Z	
Organelles (one complete allele)	Mitochondrial genome: 19.95 kb	

* Assembly metric benchmarks are adapted from Rhie *et al.* (2021) and the Earth BioGenome Project Report on Assembly Standards September 2024. ** BUSCO scores based on the vertebrata_odb10 BUSCO set using version 5.4.3. C = complete [S = single copy, D = duplicated], F = fragmented, M = missing, n = number of orthologues in comparison.

**Table 3.** Chromosomal pseudomolecules in both haplotypes of the genome assembly of *Calonectris borealis*, bCalBor7.

Haplotype 1				Haplotype 2			
INSDC accession	Name	Length (Mb)	GC%	INSDC accession	Name	Length (Mb)	GC%
OZ077811.1	1	221.11	41	OZ077772.1	1	220.95	41
OZ077812.1	2	171.79	41	OZ077773.1	2	171.96	41
OZ077813.1	3	129.33	41	OZ077774.1	3	129.08	41
OZ077814.1	4	86.02	40.5	OZ077775.1	4	86.67	40.5
OZ077815.1	5	52.55	42.5	OZ077776.1	5	52.33	42
OZ077817.1	6	44.95	42	OZ077777.1	6	44.82	42
OZ077816.1	7	44.82	42.5	OZ077778.1	7	44.93	42.5
OZ077818.1	8	40.15	42.5	OZ077779.1	8	39.28	42
OZ077819.1	9	30.47	43	OZ077780.1	9	30.59	43
OZ077820.1	10	27.21	44	OZ077781.1	10	27.8	44
OZ077821.1	11	26.25	43.5	OZ077782.1	11	26.13	43.5
OZ077823.1	12	25.22	43	OZ077783.1	12	25.06	43
OZ077822.1	13	24.83	43.5	OZ077784.1	13	25.1	43.5
OZ077825.1	14	23.81	43	OZ077785.1	14	22.8	43
OZ077824.1	15	23.79	45	OZ077786.1	15	23.46	45
OZ077826.1	16	21.86	45.5	OZ077787.1	16	21.99	45.5
OZ077827.1	17	18.31	46	OZ077788.1	17	18.21	46
OZ077828.1	18	17.53	46	OZ077789.1	18	17.48	46
OZ077829.1	19	15.47	47	OZ077790.1	19	15.47	47
OZ077830.1	20	14.89	47	OZ077791.1	20	14.91	47
OZ077831.1	21	13.17	48	OZ077792.1	21	13.02	48
OZ077832.1	22	11.06	50.5	OZ077793.1	22	10.99	51
OZ077833.1	23	9.97	47	OZ077794.1	23	9.9	46.5
OZ077834.1	24	9.17	49	OZ077795.1	24	9.0	49
OZ077835.1	25	8.84	50	OZ077796.1	25	8.81	50
OZ077836.1	26	8.73	51.5	OZ077797.1	26	7.82	51.5
OZ077837.1	27	7.81	49	OZ077798.1	27	7.02	48.5
OZ077838.1	28	6.86	53	OZ077799.1	28	6.7	52.5
OZ077840.1	29	5.1	57	OZ077800.1	29	4.73	57.5
OZ077839.1	30	4.59	56.5	OZ077801.1	30	4.98	57.5
OZ077841.1	31	1.79	62.5	OZ077802.1	31	1.5	63.5
OZ077843.1	32	1.34	62.5	OZ077803.1	32	1.15	64
OZ077842.1	33	1.2	62	OZ077804.1	33	1.27	62.5
OZ077846.1	34	0.96	68	OZ077805.1	34	0.72	67
OZ077844.1	35	0.93	63.5	OZ077806.1	35	0.83	64
OZ077845.1	36	0.73	64.5	OZ077807.1	36	0.74	63.5
OZ077847.1	37	0.71	66.5	OZ077808.1	37	0.6	66.5
OZ077848.1	38	0.63	62.5	OZ077809.1	38	0.55	63
OZ077849.1	39	0.51	65.5	OZ077810.1	39	0.47	65
OZ122107.1	W	56.73	44.5				
OZ122106.1	Z	87.72	41				
OZ077850.1	MT	0.02	42.5				

Haplotype 2 was also assembled to chromosome level, with a total length of 1,211.47 Mb in 281 sequence scaffolds, with a scaffold N50 of 86.7 Mb ( Table 2). Both sex chromosomes were submitted as part of the haplotype 1 assembly, following the INSDC convention.

The estimated Quality Value (QV) of the haplotype 1 assembly is 66.4 with *k*-mer completeness of 100.0%. For haplotype 2, the estimated Quality Value (QV) of the final assembly is 66.6 with *k*-mer completeness of 100.0%. BUSCO (v5.4.3) analysis using the vertebrata_odb10 reference set ( *n* = 8,338), indicated a completeness of 97.3% (single = 95.9%, duplicated = 1.4%) for haplotype 1, while the haplotype 2 assembly has a BUSCO v5.4.3 completeness of 93.0% (single = 92.7%, duplicated = 0.3%).

Metadata for specimens, BOLD barcode results, spectra estimates, sequencing runs, contaminants and pre-curation assembly statistics are given at https://links.tol.sanger.ac.uk/species/1323832.

## Methods

### Sample acquisition

Blood was sampled from an adult female Cory’s shearwater (specimen ID SAN00002916; ToLID bCalBor7) from the colony of Veneguera, Gran Canaria (Spain) on 2022-10-13. The blood sample was collected from the bird’s leg using a 1 ml syringe and stored in a 2 ml vial with 100% ethanol in a refrigerator. The sample was transferred to a –80 °C freezer upon arrival at the Universitat de Barcelona, five days later. The specimen was collected and identified by Jacob González-Solís.

### Nucleic acid extraction

The workflow for high molecular weight (HMW) DNA extraction at the Wellcome Sanger Institute (WSI) Tree of Life Core Laboratory includes a sequence of core procedures: sample preparation and homogenisation, DNA extraction, fragmentation and purification. Detailed protocols are available on protocols.io ( Denton *et al.*, 2023c).

The bCalBor7 blood sample was prepared for DNA extraction on dry ice ( Jay *et al.*, 2023), and was homogenised using a PowerMasher II tissue disruptor ( Denton *et al.*, 2023a). HMW DNA was extracted using the Manual Nucleated Blood Nanobind ^®^ protocol ( Denton *et al.*, 2023b). DNA was sheared into an average fragment size of 12–20 kb in a Megaruptor 3 system ( Bates *et al.*, 2023). Sheared DNA was purified by solid-phase reversible immobilisation, using AMPure PB beads to eliminate shorter fragments and concentrate the DNA ( Oatley *et al.*, 2023). The concentration of the sheared and purified DNA was assessed using a Nanodrop spectrophotometer and Qubit Fluorometer using the Qubit dsDNA High Sensitivity Assay kit. Fragment size distribution was evaluated by running the sample on the FemtoPulse system.

### Hi-C preparation

The bCalBor7 blood sample was processed at the WSI Scientific Operations core, using the Arima-HiC v2 kit. In brief, frozen tissue (stored at –80 °C) was fixed, and the DNA crosslinked using a TC buffer with 22% formaldehyde. After crosslinking, the tissue was homogenised using the Diagnocine Power Masher-II and BioMasher-II tubes and pestles. Following the kit manufacturer's instructions, crosslinked DNA was digested using a restriction enzyme master mix. The 5’-overhangs were then filled in and labelled with biotinylated nucleotides and proximally ligated. An overnight incubation was carried out for enzymes to digest remaining proteins and for crosslinks to reverse. A clean up was performed with SPRIselect beads prior to library preparation.

### Library preparation and sequencing

Library preparation and sequencing were performed at the WSI Scientific Operations core. Pacific Biosciences HiFi circular consensus DNA sequencing libraries were prepared using the PacBio Express Template Preparation Kit v2.0 (Pacific Biosciences, California, USA) as per the manufacturer’s instructions. The kit includes the reagents required for removal of single-strand overhangs, DNA damage repair, end repair/A-tailing, adapter ligation, and nuclease treatment. Library preparation also included a library purification step using 0.8X AMPure PB beads and a size selection step to remove templates < 3 kb using AMPure PB modified SPRI. Samples were sequenced using the Sequel IIe system (Pacific Biosciences, California, USA). The concentration of the library loaded onto the Sequel IIe was within the manufacturer's recommended loading concentration range of 40–100 pM. The SMRT link software, a PacBio web-based end-to-end workflow manager, was used to set-up and monitor the run, as well as perform primary and secondary analysis of the data upon completion.

Pacific Biosciences SMRTbell libraries were also constructed using the Revio HiFi prep kit, according to the manufacturers’ instructions, and DNA sequencing was performed by the Scientific Operations core at the WSI on a Pacific Biosciences Revio instrument.

For Hi-C library preparation, DNA was fragmented to a size of 400 to 600 bp using a Covaris E220 sonicator. The DNA was then enriched, barcoded, and amplified using the NEBNext Ultra II DNA Library Prep Kit following manufacturers’ instructions. The Hi-C sequencing was performed using paired-end sequencing with a read length of 150 bp on an Illumina NovaSeq X instrument.

### Genome assembly, curation and evaluation

***Assembly***

The HiFi reads were first assembled using Hifiasm ( Cheng *et al.*, 2021), which was run in the Hi-C phasing mode. The Hi-C reads were mapped to the primary contigs using bwa-mem2 ( Vasimuddin *et al.*, 2019). The contigs were further scaffolded using the provided Hi-C data ( Rao *et al.*, 2014) in YaHS ( Zhou *et al.*, 2023) using the --break option. The scaffolded assemblies were evaluated using Gfastats ( Formenti *et al.*, 2022), BUSCO ( Manni *et al.*, 2021) and MERQURY.FK ( Rhie *et al.*, 2020).

The mitochondrial genome was assembled using MitoHiFi ( Uliano-Silva *et al.*, 2023), which runs MitoFinder ( Allio *et al.*, 2020) and uses these annotations to select the final mitochondrial contig and to ensure the general quality of the sequence.

***Assembly curation***

The assembly was decontaminated using the Assembly Screen for Cobionts and Contaminants (ASCC) pipeline (article in preparation). Flat files and maps used in curation were generated in TreeVal ( Pointon *et al.*, 2023). Manual curation was primarily conducted using PretextView ( Harry, 2022), with additional insights provided by JBrowse2 ( Diesh *et al.*, 2023) and HiGlass ( Kerpedjiev *et al.*, 2018). Scaffolds were visually inspected and corrected as described by Howe *et al.* (2021). Any identified contamination, missed joins, and mis-joins were corrected, and duplicate sequences were tagged and removed. The curation process is documented at https://gitlab.com/wtsi-grit/rapid-curation (article in preparation).

***Evaluation of the final assembly***

The final assembly was post-processed and evaluated using the three Nextflow ( Di Tommaso *et al.*, 2017) DSL2 pipelines: sanger-tol/readmapping ( Surana *et al.*, 2023a), sanger-tol/genomenote ( Surana *et al.*, 2023b), and sanger-tol/blobtoolkit ( Muffato *et al.*, 2024). The readmapping pipeline aligns the Hi-C reads using bwa-mem2 ( Vasimuddin *et al.*, 2019) and combines the alignment files with SAMtools ( Danecek *et al.*, 2021). The genomenote pipeline converts the Hi-C alignments into a contact map using BEDTools ( Quinlan & Hall, 2010) and the Cooler tool suite ( Abdennur & Mirny, 2020). The contact map is visualised in HiGlass ( Kerpedjiev *et al.*, 2018). This pipeline also generates assembly statistics using the NCBI datasets report ( Sayers *et al.*, 2024), computes *k*-mer completeness and QV consensus quality values with FastK and MERQURY.FK, and runs BUSCO ( Manni *et al.*, 2021) to assess completeness.

The blobtoolkit pipeline is a Nextflow port of the previous Snakemake Blobtoolkit pipeline ( Challis *et al.*, 2020). It aligns the PacBio reads in SAMtools and minimap2 ( Li, 2018) and generates coverage tracks for regions of fixed size. In parallel, it queries the GoaT database ( Challis *et al.*, 2023) to identify all matching BUSCO lineages to run BUSCO ( Manni *et al.*, 2021). For the three domain-level BUSCO lineages, the pipeline aligns the BUSCO genes to the UniProt Reference Proteomes database ( Bateman *et al.*, 2023) with DIAMOND ( Buchfink *et al.*, 2021) blastp. The genome is also split into chunks according to the density of the BUSCO genes from the closest taxonomic lineage, and each chunk is aligned to the UniProt Reference Proteomes database with DIAMOND blastx. Genome sequences without a hit are chunked with seqtk and aligned to the NT database with blastn ( Altschul *et al.*, 1990). The blobtools suite combines all these outputs into a blobdir for visualisation.

The genome assembly and evaluation pipelines were developed using nf-core tooling ( Ewels *et al.*, 2020) and MultiQC ( Ewels *et al.*, 2016), relying on the Conda package manager, the Bioconda initiative ( Grüning *et al.*, 2018), the Biocontainers infrastructure ( da Veiga Leprevost *et al.*, 2017), as well as the Docker ( Merkel, 2014) and Singularity ( Kurtzer *et al.*, 2017) containerisation solutions.

Table 4 contains a list of relevant software tool versions and sources.

**Table 4.** Software tools: versions and sources.

Software tool	Version	Source
BEDTools	2.30.0	https://github.com/arq5x/bedtools2
BLAST	2.14.0	ftp://ftp.ncbi.nlm.nih.gov/blast/executables/blast+/
BlobToolKit	4.3.7	https://github.com/blobtoolkit/blobtoolkit
BUSCO	5.4.3 and 5.5.0	https://gitlab.com/ezlab/busco
bwa-mem2	2.2.1	https://github.com/bwa-mem2/bwa-mem2
Cooler	0.8.11	https://github.com/open2c/cooler
DIAMOND	2.1.8	https://github.com/bbuchfink/diamond
fasta_windows	0.2.4	https://github.com/tolkit/fasta_windows
FastK	427104ea91c78c3b8b8b49f1a7d6bbeaa869ba1c	https://github.com/thegenemyers/FASTK
Gfastats	1.3.6	https://github.com/vgl-hub/gfastats
GoaT CLI	0.2.5	https://github.com/genomehubs/goat-cli
Hifiasm	0.19.8-r603	https://github.com/chhylp123/hifiasm
HiGlass	44086069ee7d4d3f6f3f0012569789ec138f42b84 aa44357826c0b6753eb28de	https://github.com/higlass/higlass
Merqury.FK	d00d98157618f4e8d1a9190026b19b471055b22e	https://github.com/thegenemyers/MERQURY.FK
MitoHiFi	3	https://github.com/marcelauliano/MitoHiFi
MultiQC	1.14, 1.17, and 1.18	https://github.com/MultiQC/MultiQC
NCBI Datasets	15.12.0	https://github.com/ncbi/datasets
Nextflow	23.04.0-5857	https://github.com/nextflow-io/nextflow
samtools	1.16.1, 1.17, and 1.18	https://github.com/samtools/samtools
sanger-tol/ascc	-	https://github.com/sanger-tol/ascc
sanger-tol/genomenote	1.1.1	https://github.com/sanger-tol/genomenote
sanger-tol/readmapping	1.2.1	https://github.com/sanger-tol/readmapping
Seqtk	1.3	https://github.com/lh3/seqtk
Singularity	3.9.0	https://github.com/sylabs/singularity
TreeVal	1.0.0	https://github.com/sanger-tol/treeval
YaHS	1.2a.2	https://github.com/c-zhou/yahs

### Wellcome Sanger Institute – Legal and Governance

The materials that have contributed to this genome note have been supplied by a Darwin Tree of Life Partner. The submission of materials by a Darwin Tree of Life Partner is subject to the **‘Darwin Tree of Life Project Sampling Code of Practice’**, which can be found in full on the Darwin Tree of Life website here. By agreeing with and signing up to the Sampling Code of Practice, the Darwin Tree of Life Partner agrees they will meet the legal and ethical requirements and standards set out within this document in respect of all samples acquired for, and supplied to, the Darwin Tree of Life Project.

Further, the Wellcome Sanger Institute employs a process whereby due diligence is carried out proportionate to the nature of the materials themselves, and the circumstances under which they have been/are to be collected and provided for use. The purpose of this is to address and mitigate any potential legal and/or ethical implications of receipt and use of the materials as part of the research project, and to ensure that in doing so we align with best practice wherever possible. The overarching areas of consideration are:

•    Ethical review of provenance and sourcing of the material

•    Legality of collection, transfer and use (national and international)

Each transfer of samples is further undertaken according to a Research Collaboration Agreement or Material Transfer Agreement entered into by the Darwin Tree of Life Partner, Genome Research Limited (operating as the Wellcome Sanger Institute), and in some circumstances other Darwin Tree of Life collaborators.

## Data Availability

European Nucleotide Archive: Calonectris borealis (Cory's shearwater). Accession number PRJEB75561; https://identifiers.org/ena.embl/PRJEB75561. The genome sequence is released openly for reuse. The *Calonectris borealis* genome sequencing initiative is part of the Darwin Tree of Life (DToL) project. All raw sequence data and the assembly have been deposited in INSDC databases. The genome will be annotated using available RNA-Seq data and presented through the Ensembl pipeline at the European Bioinformatics Institute. Raw data and assembly accession identifiers are reported in Table 1 and Table 2.

## Author information

Members of the Wellcome Sanger Institute Tree of Life Management, Samples and Laboratory team are listed here: https://doi.org/10.5281/zenodo.12162482.

Members of Wellcome Sanger Institute Scientific Operations: Sequencing Operations are listed here: https://doi.org/10.5281/zenodo.12165051.

Members of the Wellcome Sanger Institute Tree of Life Core Informatics team are listed here: https://doi.org/10.5281/zenodo.12160324.

Members of the Tree of Life Core Informatics collective are listed here: https://doi.org/10.5281/zenodo.12205391.

Members of the Darwin Tree of Life Consortium are listed here: https://doi.org/10.5281/zenodo.4783558.
