# Supplementary material for: The genome sequence of Cory’s shearwater, Calonectris borealis (Cory, 1881)
Source: Wellcome Open Res. 2024 Nov 12;9:678. [Version 1] doi: 10.12688/wellcomeopenres.23354.1 (PMC11806253; doi:10.12688/wellcomeopenres.23354.1)
Supplement: Peer review history

# Reviewer report: Reviewer response for version 1

**DOI:** 10.21956/wellcomeopenres.25742.r116398

**Published:** 2025-02-07

**Authors:** Hernan E Morales

**Affiliations:** 1 University of Copenhagen, Copenhagen, Denmark

The authors report the first chromosome-level genome assembly of Cory’s shearwater ( *Calonectris borealis*) using PacBio HiFi and scaffolded with Hi-C data.  Haplotype 1 is scaffolded into 41 chromosomal pseudomolecules, including the Z and W sex chromosomes, while haplotype 2 consists of 39 autosomes. BUSCO completeness scores indicate high-quality assembly, with 97.3% and 93.0% completeness for haplotypes 1 and 2, respectively.

 This genome assembly has potential applications in seabird migration studies, conservation genomics, and population genetics. The manuscript is well-structured, with detailed methods and a clear presentation of results.

 I found the motivation for working on this "model species for studies on movement ecology and seabird migration" very interesting. The manuscript could benefit from further details. For example, if the authors were to expand this section a little bit explaining their hypotheses and questions regarding the migratory behavior of Cory’s shearwater and putting them in the context of previous work on other migratory species. 

 Haplotype resolution: while the assembly includes two haplotypes, there is limited discussion on potential phasing errors or inconsistencies in haplotype separation. Clarifying how phasing was validated (e.g., through read-depth analysis or alternative scaffolding approaches) would strengthen confidence in the assembly.

 Contamination screening: the report mentions the use of ASCC for decontamination, but there is no explicit mention of the contamination threshold applied or whether any microbial/host contamination was detected. More details could help 

 Hi-C processing: the Hi-C library preparation is mentioned, but there is no description of the depth of sequencing required for proper chromosomal scaffolding. Including estimates of required coverage for Hi-C data would enhance reproducibility and help others

Are sufficient details of methods and materials provided to allow replication by others?

Partly

Is the rationale for creating the dataset(s) clearly described?

Yes

Are the datasets clearly presented in a useable and accessible format?

Yes

Are the protocols appropriate and is the work technically sound?

Yes

Reviewer Expertise:

Evolutionary and conservation genomics

I confirm that I have read this submission and believe that I have an appropriate level of expertise to confirm that it is of an acceptable scientific standard.

# Reviewer report: Reviewer response for version 1

**DOI:** 10.21956/wellcomeopenres.25742.r113386

**Published:** 2024-12-11

**Authors:** Chen Siang Ng

**Affiliations:** 1 Institute of Molecular and Cellular Biology & Department of Life Science, National Tsing Hua University, Hsinchu, Taiwan

This article presents the first high-quality, chromosome-level genome assembly for Cory’s shearwater ( *Calonectris borealis*), a pelagic seabird widely distributed across the Atlantic Ocean. Based on a female specimen, the assembly includes two haplotypes with respective total lengths of 1.37 Gb and 1.21 Gb, scaffolded using PacBio HiFi sequencing and Hi-C data. The genome assembly achieves high completeness, as indicated by BUSCO scores (97.3% and 93.0% for haplotypes 1 and 2, respectively). This dataset has potential applications in studies of seabird migration, population genetics, and conservation.

 Comments:

 1) The manuscript should address whether a voucher specimen was deposited and provide details on its documentation for future taxonomic reference. Voucher specimens are critical for ensuring the reproducibility and long-term utility of genomic studies, as highlighted in the following publication:

 Buckner JC, Sanders RC, Faircloth BC, Chakrabarty P. (2021). The critical importance of vouchers in genomics. Elife, 10, e68264. doi: 10.7554/eLife.68264.

 Including information on voucher specimens and their accessibility would enhance the reliability and reproducibility of the study, particularly for future taxonomic comparisons.

 2)  To strengthen the background and discussion, the authors should cite and discuss the following papers, which are highly relevant to the genomic and ecological aspects of migratory seabirds:

 i) A recent genomic paper on migratory seabirds:

 Herman RW, Clucas G, Younger J, Bates J, Robinson B, Reddy S, Stepanuk J, O'Brien K, Veeramah K, Lynch HJ. (2024). Whole genome sequencing reveals stepping-stone dispersal buffered against founder effects in a range-expanding seabird. Molecular Ecology, 33(6), e17282. doi: 10.1111/mec.17282.

 This paper provides insights into dispersal mechanisms and the role of genomic approaches in understanding seabird migration and population structure, making it highly relevant to the current study.

 ii) A review paper on range expansions:

 Excoffier, L., Foll, M., & Petit, R. J. (2009). Genetic consequences of range expansions. Annual Review of Ecology, Evolution, and Systematics, 40, 481–501.

 This comprehensive review discusses the genetic consequences of range expansions, providing a framework for interpreting genomic data in the context of population dynamics and evolutionary processes.

 iii) Population genomics in seabirds:

 Friesen, V. L., Burg, T. M., & McCoy, K. D. (2007). Mechanisms of population differentiation in seabirds. Molecular Ecology, 16(9), 1765–1785.

 Herman, R. W., Winger, B. M., Dittmann, D. L., & Harvey, M. G. (2022). Fine-scale population genetic structure and barriers to gene flow in a widespread seabird (Ardenna pacifica). Biological Journal of the Linnean Society, 137(1), 125–136.

 These papers provide valuable insights into the mechanisms of population differentiation, genetic structure, and barriers to gene flow in seabirds, which are directly relevant to the current study.

 iv) Ecological and environmental influences on seabird populations:

 Barbraud, C., & Delord, K. (2021). Selection against immigrants in wild seabird populations. Ecology Letters, 24(1), 84–93.

 Emmerson, L., & Southwell, C. (2022). Environment-triggered demographic changes cascade and compound to propel a dramatic decline of an Antarctic seabird metapopulation. Global Change Biology, 28(24), 7234–7249.

 These studies highlight the impact of environmental and demographic factors on seabird populations, offering a broader ecological context for interpreting the genomic findings.

 v) Whole genome analyses of seabird populations:

 Kersten, O., Star, B., Leigh, D. M., Anker-Nilssen, T., Strøm, H., Danielsen, J., Descamps, S., Erikstad, K. E., Fitzsimmons, M. G., Fort, J., Hansen, E. S., Harris, M. P., Irestedt, M., Kleven, O., Mallory, M. L., Jakobsen, K., & Boessenkool, S. (2021). Complex population structure of the Atlantic puffin revealed by whole genome analyses. Communications Biology, 4(1), 922.

 This study demonstrates the power of whole-genome analyses in uncovering complex population structures, which is relevant for interpreting gene flow and differentiation patterns in Cory’s shearwater.

 By incorporating these references, the manuscript will provide a richer and more comprehensive scientific context, enhancing its discussion of migration, population genomics, and conservation genetics in seabirds.

Are sufficient details of methods and materials provided to allow replication by others?

Yes

Is the rationale for creating the dataset(s) clearly described?

Yes

Are the datasets clearly presented in a useable and accessible format?

Yes

Are the protocols appropriate and is the work technically sound?

Yes

Reviewer Expertise:

Evolutionary genomics, evolutionary ornithology, evo-devo

I confirm that I have read this submission and believe that I have an appropriate level of expertise to confirm that it is of an acceptable scientific standard.
